# Supplementary figures and images for: Environment and weight class linked to skin microbiome structure of juvenile Eastern hellbenders (Cryptobranchus alleganiensis alleganiensis) in human care
Source: PLoS One. 2025 Oct 1;20(10):e0319317. doi: 10.1371/journal.pone.0319317 (PMC12488022; doi:10.1371/journal.pone.0319317)

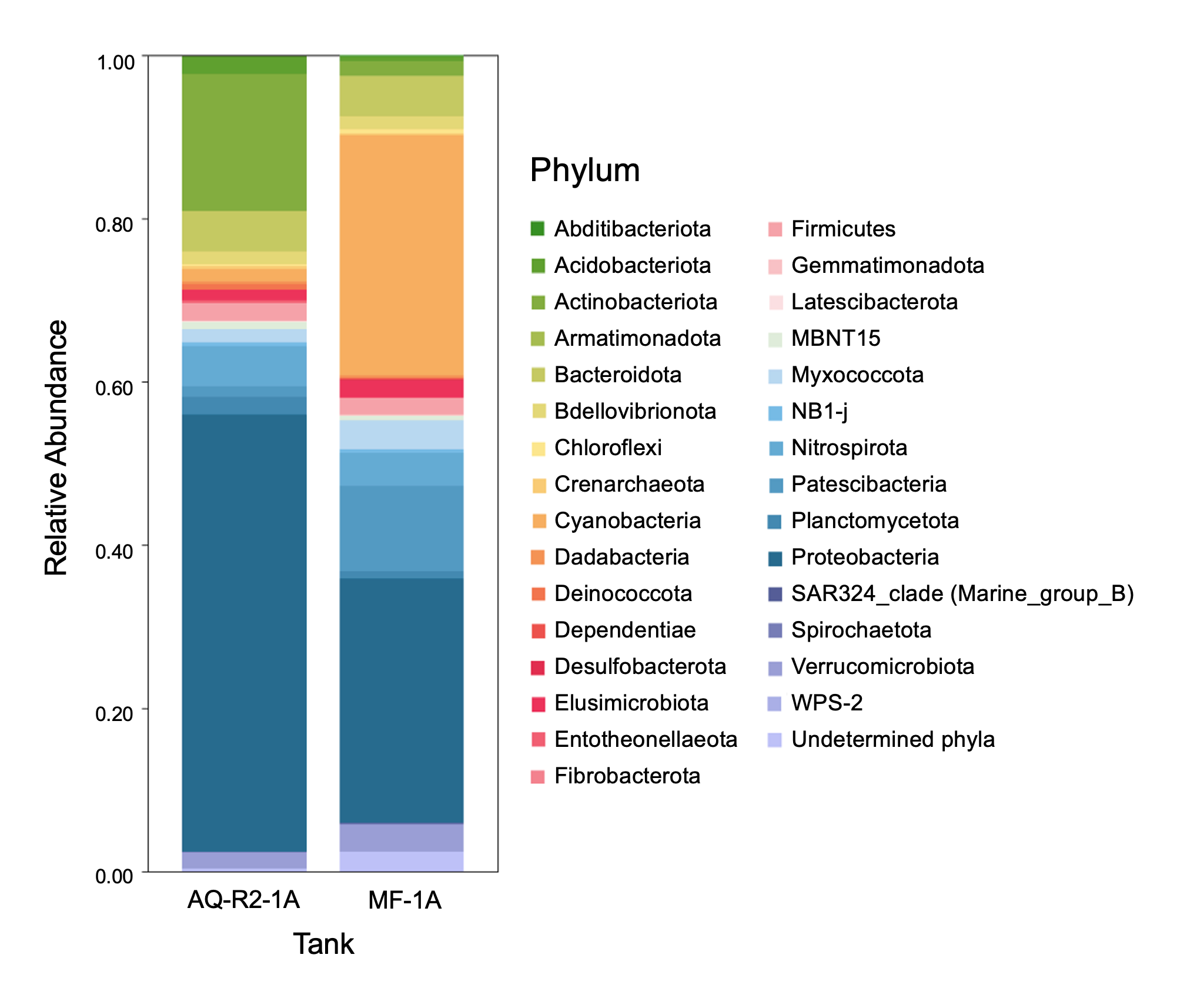

Supplement: S1 Fig — (TIF) [file pone.0319317.s001.tif]

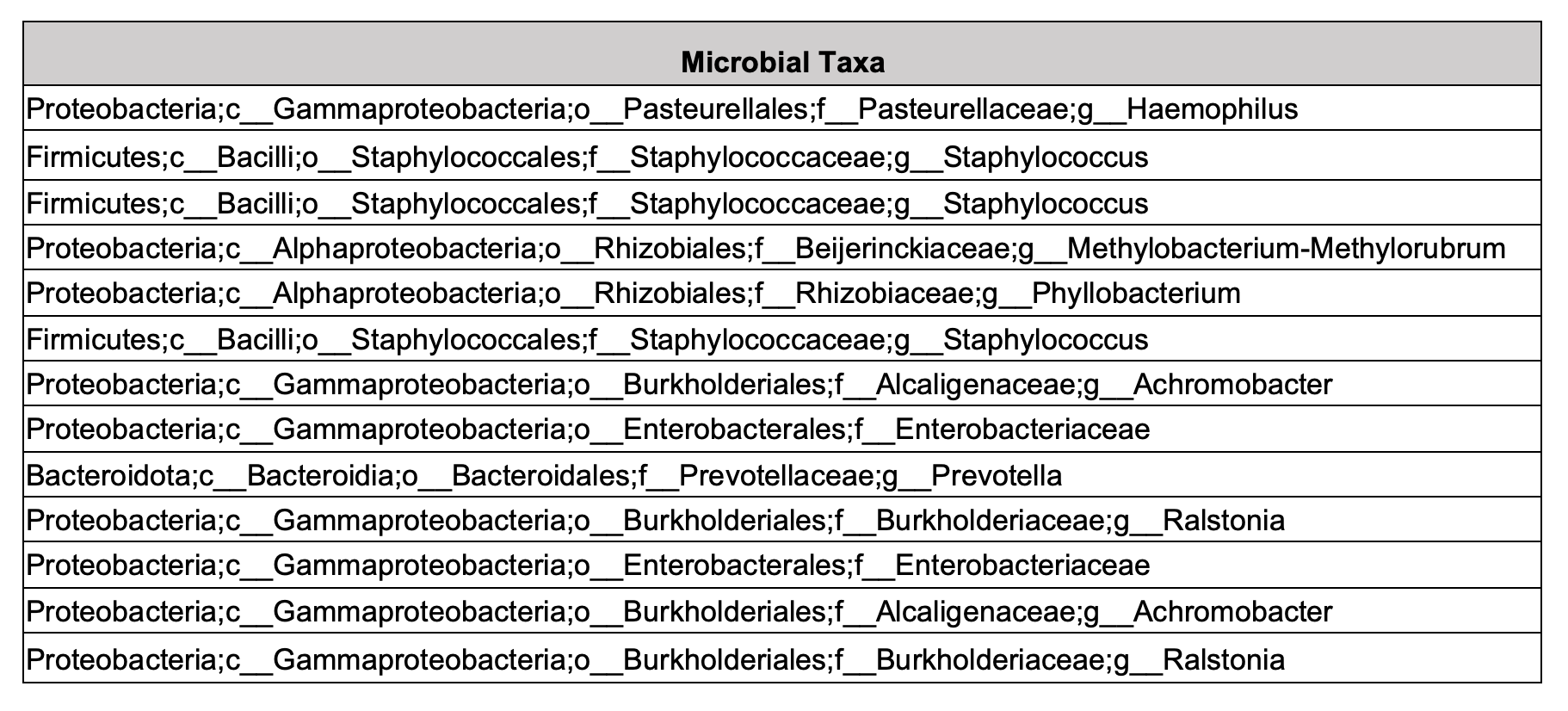

Supplement: S1 Table — A total of 13 taxa were identified as putative contaminants and bioinformatically removed from further analysis. (TIF) [file pone.0319317.s002.tif]

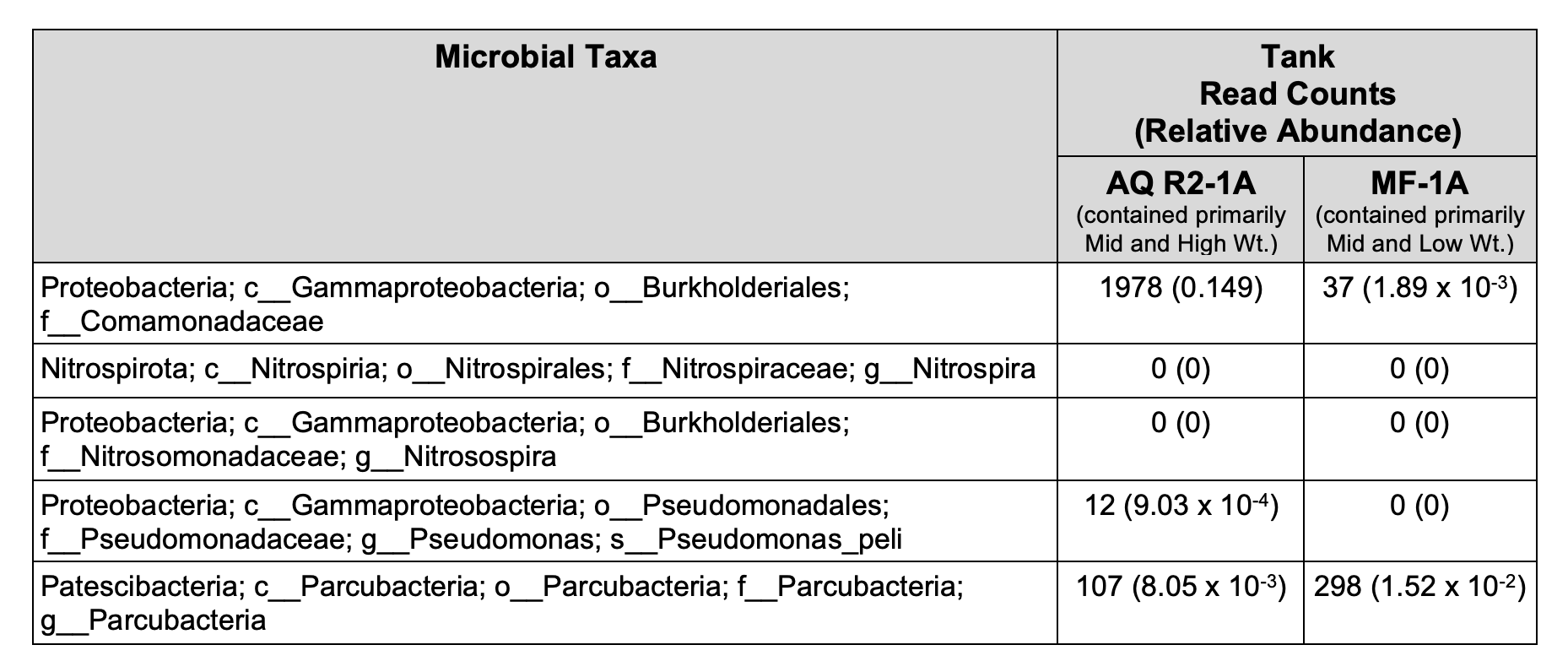

Supplement: S2 Table — Three of the five microbial taxa that were differentially abundant by weight class were also detected at variable abundances in water samples collected from tanks AQ R2-1A and MF-1A. (TIF) [file pone.0319317.s003.tif]

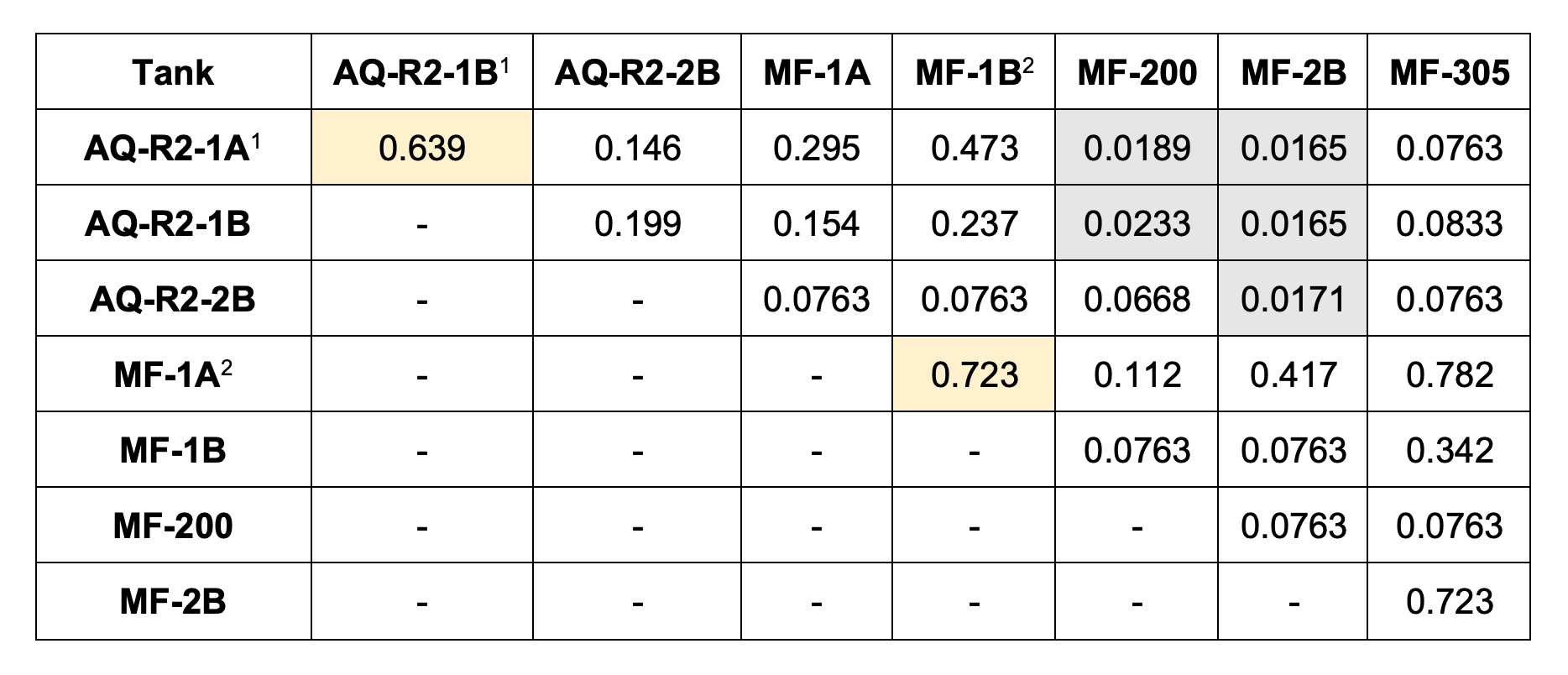

Supplement: S3 Table — Hellbender skin microbial diversity differed significantly by tank (Shannon Diversity Index: p = 2.27 x 10^−4^). Significant pairwise comparisons (p < 0.05, shaded in gray) revealed that hellbenders in tanks located in different buildings and maintained on different water systems were more likely to differ in microbial diversity than hellbenders located in tanks inside the same building/ room or on a shared water system. Superscripts indicate tanks with a shared water and filtration system (shaded in yellow). These tanks were the most similar in terms of microbial diversity. (TIF) [file pone.0319317.s004.tif]

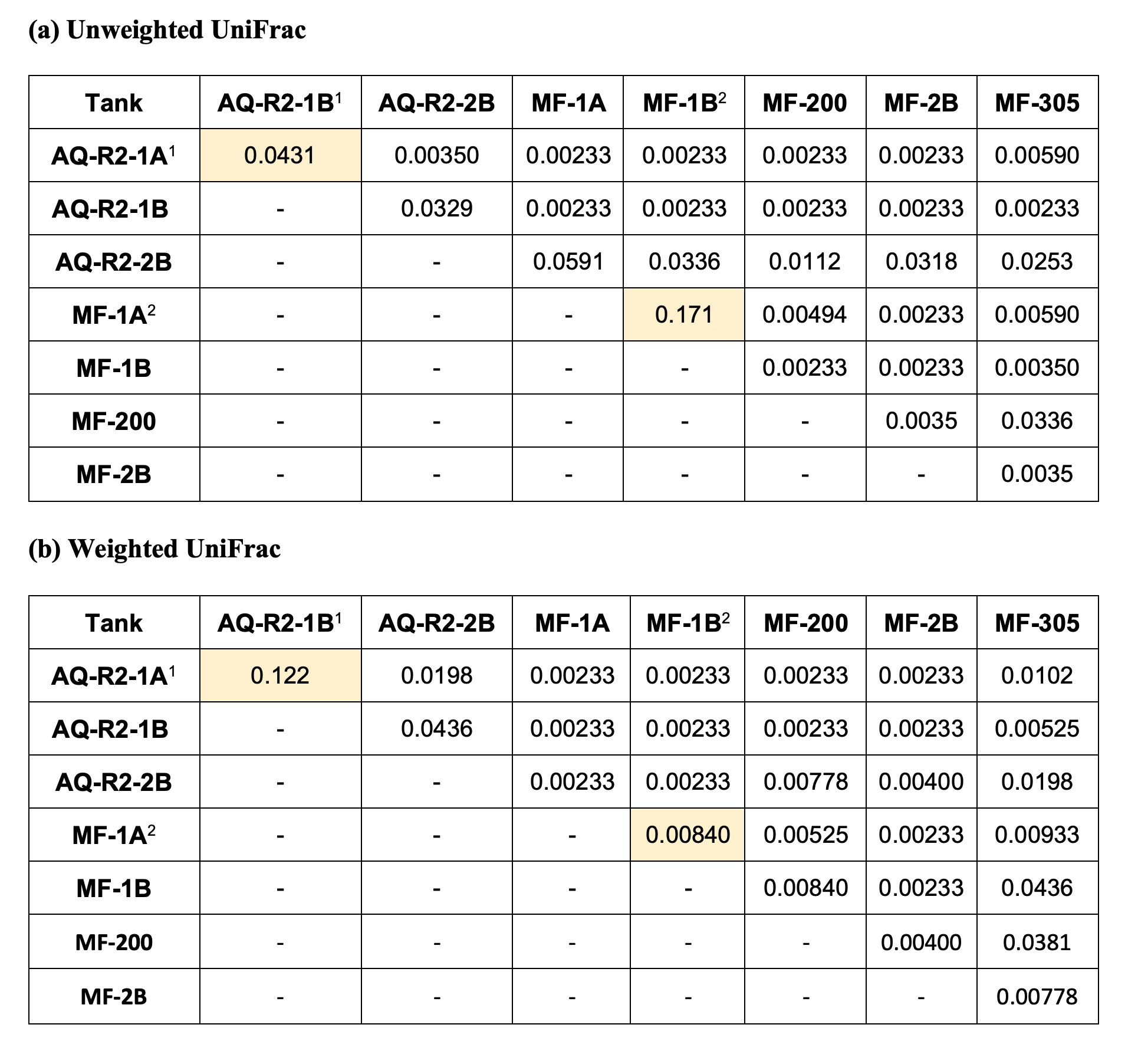

Supplement: S4 Table — Hellbender skin microbial diversity differed significantly by tank based on (a) unweighted and (b) weighted UniFrac distance metrics (p < 0.001). The majority of pairwise comparisons were significantly different (p < 0.05) indicating significant microbial composition differences by tank. Hellbenders in tanks that shared a water and filtration system (shaded in yellow) generally had more similar skin microbiota. (TIF) [file pone.0319317.s005.tif]

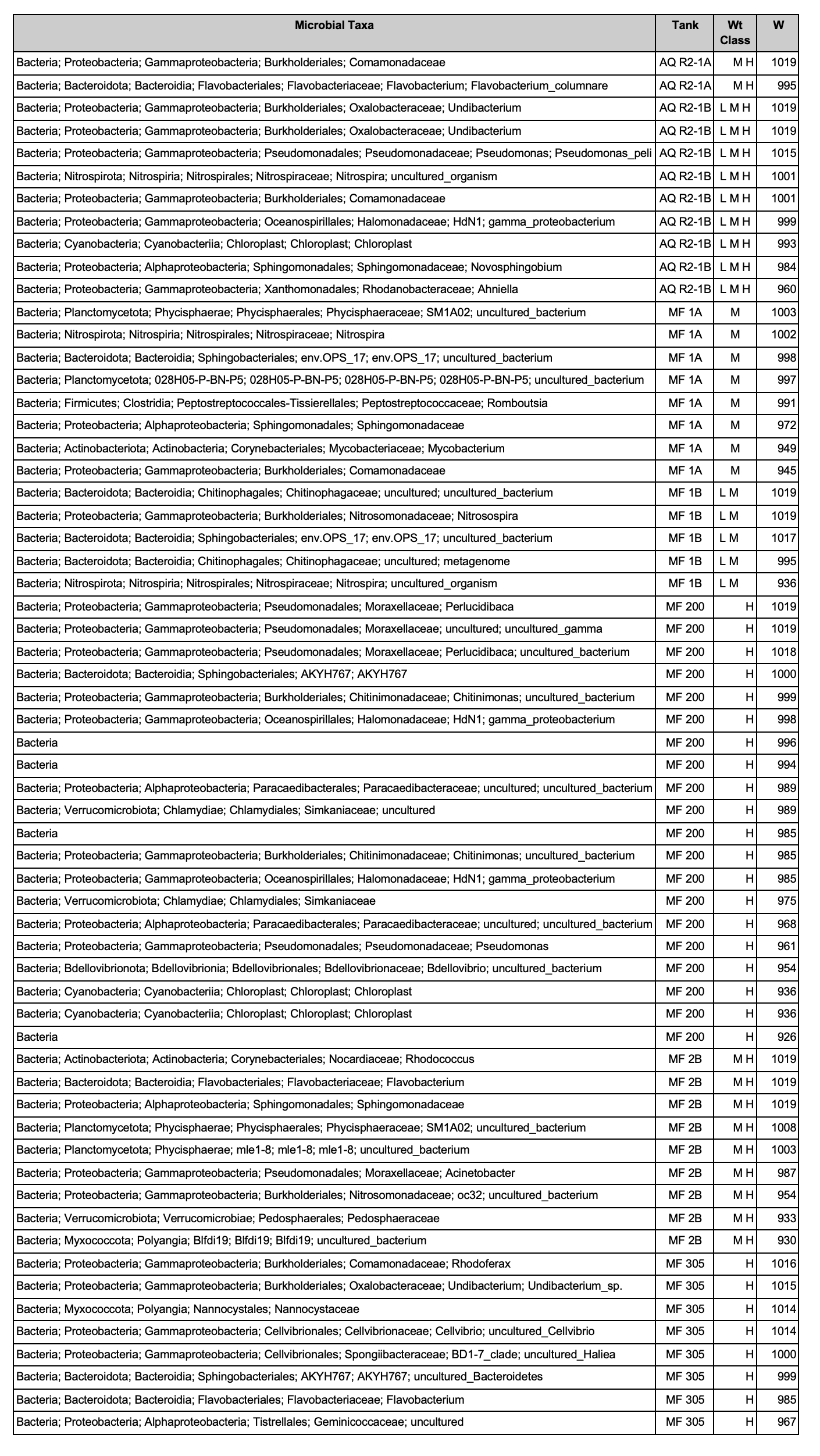

Supplement: S5 Table — A total of 61 differentially abundant taxa (ANCOM) by tank were identified among tanks holding three or more hellbenders. Column labeled “Tank” indicates the tank in which the taxa was present in highest abundance. Column labeled “Wt Class” indicates if animals contained Low (L), Mid (M), and/or High (H) weight class animals. (TIF) [file pone.0319317.s006.tif]
