# Supplementary material for: Environment and weight class linked to skin microbiome structure of juvenile Eastern hellbenders (Cryptobranchus alleganiensis alleganiensis) in human care
Source: PLoS One. 2025 Oct 1;20(10):e0319317. doi: 10.1371/journal.pone.0319317 (PMC12488022; doi:10.1371/journal.pone.0319317)
Supplement: Attachment — Submitted filename: Response to Reviewers.docx [file pone.0319317.s008.docx]

July 20, 2025

Alexandre Ribeiro da Silva

Acadmic Editor, PLOS ONE

Dear Dr. Ribeiro da Silva:

Thank you for allowing us to submit a revised manuscript titled “Environment and weight class linked to skin microbiome structure of juvenile Eastern hellbenders (*Cryptobranchus alleganiensis alleganiensis*) in human care” for further consideration for publication in PLOS ONE. We are grateful to you and the reviewers for your efforts and time to review our manuscript. We have carefully revised the manuscript to address the questions and suggestions raised. Below is a point-by-point response (blue text) to the comments and suggestions provided June 2, 2025.

**Editor and Journal Requirements:**

1. Please ensure that your manuscript meets PLOS ONE's style requirements, including those for file naming. The PLOS ONE style templates can be found at <https://journals.plos.org/plosone/s/file?id=wjVg/PLOSOne_formatting_sample_main_body.pdf> and <https://journals.plos.org/plosone/s/file?id=ba62/PLOSOne_formatting_sample_title_authors_affiliations.pdf>
   - **We have edited the manuscript format to meet the style requirements and corrected the level of all headings.**
2. Please note that funding information should not appear in any section or other areas of your manuscript. We will only publish funding information present in the Funding Statement section of the online submission form. Please remove any funding-related text from the manuscript.
   - **We have edited the Acknowledgements to remove funding information.**
3. Please review your reference list to ensure that it is complete and correct. If you have cited papers that have been retracted, please include the rationale for doing so in the manuscript text, or remove these references and replace them with relevant current references. Any changes to the reference list should be mentioned in the rebuttal letter that accompanies your revised manuscript. If you need to cite a retracted article, indicate the article’s retracted status in the References list and also include a citation and full reference for the retraction notice.
   - **We have edited the reference list order only to align with the edits made in response to reviewer suggestions.**

**Reviewer #1: This is overall a neatly written paper with clear value to hellbender husbandry and reintroduction efforts. A few suggestions are as follows:**

- The organization of the introduction seems like it would benefit from some tweaking. I would move the section about the importance of amphibian skin earlier in the paper to before hellbenders are introduced. That way you begin broadly (amphibians are important and declining, their skin and microbiota is an important part of their health) and then narrow down (hellbenders are a unique amphibian species of conservation concern, we're facing these challenges with reintroduction, skin may be the key)
  - **Thank you for the suggestion. We have edited the introduction as suggested to broadly introduce the importance of amphibian skin and the microbiota and then provide specific information about Eastern hellbenders and their current conservation status.**
- I would like more information about the eggs origins. How many unique clutches are they harvested from? Are they all from the same river reach? It seems that these could influence microbiome and overall health as well.
  - **All eggs were collected from a single location and clutch during a single collection event. We have added this information to clarify these important details.**
    - **Materials and methods (Line 110): “In September 2017, the CZA received eggs collected from a single location and clutch during a single collection event from an undisclosed site in Ohio.”**
- Did you collect any data on the skin health of these guys? Given the discussions of lesions, I was curious if any of these individuals presented lesions and if you assessed differences in microbiome as it related to lesion presence or absence.
  - **In this study, we focused on animals with grossly normal skin and this was verified at the time of sampling. We feel this helped to assess the factors of tank location and weight class while focusing on animals with grossly normal skin condition. We have added this information to clarify this important detail in the Introduction objectives and in the Materials and methods.**
- Line 84--This might better read "An organism’s habitat, including the soil, water, plants, and other animals to which they may be exposed, are important influences on a host."
  - **Thank you for the suggestion. We have incorporated the edit as proposed.**
- Line 275 **--**What software and packages were used for statistical analysis?
  - **We have clarified this information in the Materials and methods to state that the adonis test (referenced by the reviewer in the Results section original line 275) was performed in QIIME2.**
    - **Materials and methods (Line 243): “We then performed multivariate adonis tests in QIIME2 to compare the effects of weight class, tank, and their interaction on skin bacterial diversity and composition.”**
- Line 313--This is a whopper of a sentence! I would break it into two.
  - **This sentence was edited into two to improve flow and clarity as suggested.**

**Reviewer #2: PLOS ONE Reviewer Report
This manuscript meets PLOS ONE’s publication criteria with scientific rigor, clarity, and relevance. I recommend acceptance pending minor revisions. This study offers valuable insights into the relationship between cutaneous microbiota, captive environmental conditions, and body condition in juvenile Eastern hellbenders—a species of high conservation priority. Methodologically, the manuscript is robust, with a strong sample size (*n* = 116) and well-chosen analytical approaches (16S rRNA sequencing, PERMANOVA, ANCOM). The findings have direct relevance for improving ex situ management practices. While the study is well executed, I recommend minor revisions to enhance clarity, statistical transparency, and ecological interpretation. The suggestions below aim to further strengthen the manuscript prior to publication. Notably, the identification of Parcubacteria as a weight-class–associated taxon presents a promising avenue for exploring host–microbiome dynamics in amphibians.**

**Recommendations for Improvement:**

1.Abstract/Introduction:

- Replace "true association" (Line 55) with more precise language like "specific association".
  - **Thank you. We have incorporated the proposed edit in the Abstract as well as in the Conclusion in a similar instance of use.**
- Better emphasize the practical applications for captive management
  - **We have added information to highlight several important examples of skin microbiome studies and experimental treatments that are directly related to management in captive settings. This addition helps provide context for the current study and improves cohesion with further additions made to the Discussion based on reviewer suggestions later.**
    - **Introduction (Lines 73-82): “Advances in characterizing and understanding various factors that influence the amphibian skin microbiota can help improve the management for amphibians under human care as well as those in free-ranging settings. Experimental studies evaluating the application of probiotic therapies to amphibian hosts have demonstrated positive health effects in response to disease [7-8]. Additionally, serial sampling of the skin microbiota of wild amphibians and those under human care can reveal changes in skin bacterial diversity over time. In some amphibian populations, depletion of skin bacterial diversity has been related to decreased normal skin defenses and increased susceptibility to skin pathogens [8]. Because amphibian skin is so integral to whole body systemic health, improved understanding of the skin microbiota can help advance the health and management of amphibians on both an individual and population level.”**

Statistical Analysis:

- PERMANOVA (Line 217): Clarify which factors were tested (e.g., weight × tank interaction) and report R² values for each factor in the results table.
  - **We have added details in the methods to indicate that we conducted independent PERMANOVAs for weight class and tank: “Microbial composition was compared across groups by weight class and separately by tank using one-way permutational multivariate analysis of variances (PERMANOVA) employing weighted and unweighted UniFrac distance matrices.”**
  - **In QIIME2 the test statistic for PERMANOVAs is a pseudo-F rather than an R². The Pseudo-F value represents the ratio of between-group variation to within-group variation, indicating the strength of separation between groups. We have now reported the pseudo-F value for all one-way PERMANOVAs (by weight class and tank) in the manuscript, and we note that we DO already provide R² values for our multivariate analysis (Adonis) by weight class and tank:**
    - **Results: “In contrast, there were significant differences in microbial composition by weight class (PERMANOVA: Unweighted UniFrac p = 0.001, pseudo-F = 2.06; Weighted UniFrac p = 0.001, pseudo-F = 2.87; Fig 3).”**
    - **Results: “We found significant differences in overall diversity and composition by tank (Shannon: p = 2.2 x 10^-4^; PERMANOVA: Unweighted UniFrac p < 0.001, pseudo-F = 2.41; Weighted UniFrac p < 0.001, pseudo-F= 5.85) (Fig 5 and S3 and S4 Tables), with more pronounced differences found in tanks that were in separate rooms or buildings with different water and filtration systems.”**
- Multivariate Analysis: Consider testing whether tank effects persist after controlling for body condition.
  - **We performed multivariate analysis in QIIME2 using the adonis function, a multi-way PERMANOVA that allowed us to test for tank effects while controlling for body condition. Tank effects persisted even after controlling for body condition. We have now clarified this in the methods and results:**
    - **Methods: “We then performed multivariate adonis tests in QIIME2 to compare the effects of weight class, tank, and their interaction on skin bacterial diversity and composition. Next, we analyzed the effects of tank alone.”**
    - **Results: “As hellbenders were generally size sorted into tanks, we performed several additional analyses to parse potentially confounding effects between weight class and tank. First, we performed a multivariate adonis analysis that allowed us to account for both weight class and tank. The results showed that both weight class and tank had significant effects on skin microbial composition (Unweighted UnifFrac: weight class R = 0.21, p < 0.001; tank R = 0.44, p < 0.001; Weighted UniFrac: weight class R = 0.25, p < 0.001; tank R = 0.57, p < 0.001), with stronger tank effects observed compared to weight effects. There was also a significant interaction between weight class and tank based on unweighted UniFrac distances (R = 0.22, p < 0.011), but not weighted UniFrac distances (R = 0.20, p < 0.11).”**

2.Methods:

- Clarify criteria for weight class divisions (≤50g, 50-80g, ≥80g)
  - **We have provided additional context and information that contributed to the development of the weight class divisions.**
    - **The updated paragraph now reads: “Animals were classified into three weight classes: Low weight (≤ 50 g), Mid weight (50-80 g), and High weight (≥ 80 g). The weight ranges for each weight class were determined based on the clinical history of this specific cohort of hellbenders, historic institutional data, and the scaled mass index (SMI) to estimate 3 categories of body condition. Specifically, hellbenders from this cohort that died approximately 6 months prior to the current study often exhibited low body weight (< 50 g) and cutaneous lesions [16]). Additionally, similarly aged animals from previous cohorts (33-34 mos old, n = 132) had a mean body weight of 83.9 g and a mean total length of 26.5 cm. The SMI was calculated as body weight x (20/total body length)^2.98^ based on institutional data from multiple previous hellbender cohorts [17]. Thus, the weight ranges and weight classes as defined above were established to categorize three distinct categories of weight with clinical relevance to both this specific study cohort and previous cohorts.”**
- Provide more details about tank maintenance protocols
  - **Additional details were added about room and tank biosecurity measures, tank maintenance and water quality monitoring, and feeding practices that add meaningful information about the study population and wider environment.**
- Expand description of contaminant removal process.
  - **We have updated this section for clarity. It now reads: “We then filtered the sequencing results for potential contaminants, or microbial taxa spuriously introduced during extraction, library preparation, or sequencing. We defined contaminants as any bacterial taxa found predominantly in low biomass negative controls (at >3% relative abundance), and at relatively low proportions (<3%) in true samples.”**

3.Results:

- Highlight that tank effects (R=0.44-0.57) were stronger than weight effects (R=0.21-0.25)
  - **Thank you. We have edited the text to better highlight this finding.**
- Emphasize Parcubacteria as the only weight-specific taxon.
  - **Thank you. We have edited the text to increase emphasis on this unique finding.**

4.Discussion:

- The observed association between Parcubacteria and low body condition in juvenile Eastern hellbenders suggests a potential role for this taxon in host health. Given its reduced genome and likely dependence on host-derived metabolites, Parcubacteria may act as a marker or contributor to microbial dysbiosis under physiological stress (Nelson & Stegen, 2015). Further discussion of its possible parasitic or opportunistic role would enhance the ecological interpretation of the findings.
  - **We have added additional detail about unique properties of *Parcubacteria*, specifically regarding the function of glycosyltransferases. We also edited the text to more pointedly discuss the potential important parasitic effects *Parcubacteria* may exert on host organisms and how hellbenders within the CZA captive-rearing program may suffer chronic cumulative stress due to the presence of *Parcubacteria*. We suggest that additional study is needed to better understand the relationship between the presence of *Parcubacteria* and overall hellbender and amphibian health to help improve the ecological context of our findings as suggested.**
    - **Discussion (Lines 398-413): “Many members of *Parcubacteria* have limited innate metabolic and respiratory capabilities and lack the ability to synthesize important compounds such as amino acids and fatty acids [34-35]. *Parcubacteria* also possess genes that code for glycosyltransferase enzymes which promote the biosynthesis of polysaccharides, polypeptides, and glycerolipids for essential metabolic functions including cell membrane synthesis and adhesion to host tissue [32]. These features suggest that many types of *Parcubacteria* rely on other organisms for essential metabolic functions and may act as parasites [35]. In hellbenders, this parasitic effect may be hindering optimal growth and contributing to increased metabolic stress. These cumulative effects can compromise systemic and skin health in Low weight animals. Ultimately these effects may contribute to the development of cutaneous abnormalities like those historically observed in captive-reared deceased juvenile hellbenders with low body condition and severe cutaneous lesions [16]. Additional studies on *Parcubacteria* are necessary and should evaluate its potential negative effect on hellbender growth and skin health. Moreover, further research is needed to determine if *Parcubacteria* may serve as a marker for skin dysbiosis in hellbenders within this system or in amphibians in other environments as well.”**
- The manuscript should also acknowledge key limitations, such as the lack of longitudinal sampling to track microbiome shifts over time or in response to health outcomes, and the absence of functional assays to test microbial pathogenicity.
  - **Thank you. We have added information about additional limitations to the discussion.**
    - **Discussion (Lines 426-438): “There are several important limitations relevant to this study. First is that we focused on characterizing the bacterial microbiota in hellbenders and did not examine microbial eukaryotes such as fungi, which are an important factor in amphibian skin disease [37-38]. Secondly, serial longitudinal sampling of the study population could have helped identify baseline patterns and potential shifts in the microbiota over time. Lastly, the absence of functional assays in this study limits our ability to characterize and evaluate potential negative and positive microbial activities and their influence on hellbender health. Despite these limitations, we identified clear factors (weight class and tank) that were significantly associated with skin bacterial composition and we were able to identify a specific bacterial taxa, *Parcubacteria*, that may reflect or influence important changes in hellbender health and skin function. These findings would not have been possible using traditional culture-based techniques. Future research should prioritize how husbandry factors and potential stressors impact the skin microbiota in hellbenders and how these potential patterns are associated with morbidity and mortality.”**
- To strengthen conservation applications, the authors are encouraged to propose practical management interventions. These include routine skin microbiome monitoring to identify early signs of dysbiosis, temporary segregation or enhanced care protocols for underweight individuals to limit microbial spread and stress, and pilot studies evaluating the probiotic potential of taxa like Pseudomonas peli, which was enriched in higher-weight animals.
  - **Thank you. We have added several suggested monitoring and management strategies to the Discussion which help highlight more pointed practical applications based on the findings of the current study.**
    - **Discussion (Lines 438-449): “In addition, we suggest that management of hellbenders under human care may be improved by implementing several strategies. For example, baseline and subsequent serial evaluation of the skin microbiota may help better characterize normal shifts in the skin microbiota over time related to various factors including diet and environmental conditions (e.g., water quality and tank properties). Serial testing can aid the identification of potential dysbiosis prior to the manifestation of obvious signs of disease. This may be especially helpful in this species which can exhibit long periods of cryptic and inactive behavior as part of their normal behaviors. With increased information about baseline and serial patterns of the skin microbiota in hellbenders, developing specific therapies (e.g., probiotic treatments) and management strategies to improve the health of hellbenders under human care would be invaluable to ex-situ conservation programs and ultimately free-ranging populations as well.”**
- These additions would not only improve the biological depth of the study but also increase its value to captive amphibian husbandry and reintroduction programs.
  - **Thank you for these meaningful suggestions. We have made notable edits as suggested that help to clarify and improve the ecological context and biological depth of the study greatly.**

Conclusion:

- The manuscript represents a valuable contribution to amphibian conservation science. With minor revisions to improve clarity and emphasize practical applications, it will be suitable for publication in PLOS ONE. The findings have immediate relevance for hellbender captive breeding programs and may inform similar conservation efforts for other amphibian species.

We sincerely thank you for your time and effort to provide detailed recommendations and comments. We have worked diligently to address each comment and feel the revised manuscript is much improved as a result. We look forward to any additional suggestions or comments and again appreciate your time and consideration.

Sincerely,

Vanessa Hale MAT, DVM, PhD

Co-Director, Infectious Disease Institute Microbial Communities Program

Associate Director, Center of Microbiome Science

Assistant Professor, Veterinary Preventive Medicine, College of Veterinary Medicine

Hale.502@osu.edu

Andrea Aplasca, DVM, MS, Dipl. ACZM

aplasca@gmail.com
